# Partial purification and properties of cyclodextrin glycosiltransferase (CGTase) from alkalophilic Bacillus species

**DOI:** 10.1186/2193-1801-1-61

**Published:** 2012-12-12

**Authors:** Marlene M Martínez Mora, Karel Hernández Sánchez, Reynaldo Villalonga Santana, Arley Pérez Rojas, Héctor L Ramírez, Juan José Torres-Labandeira

**Affiliations:** 1 Center for Enzyme Technology, University of Matanzas, Matanzas, C.P., 44740 Cuba; 2 Department of Analytical Chemistry, Faculty of Chemistry, Complutense University of Madrid, Madrid, Spain; 3 Center for Environmental Studies, University of Matanzas, Matanzas, C.P., 44740 Cuba; 4 Department of Pharmacy and Pharmaceutical Technology, University of Santiago de Compostela, Faculty of Pharmacy, Santiago de Compostela, 15782 Spain

**Keywords:** Cyclodextrins glucanotransferase, Alkalophilic *Bacillus* sp., Enzyme purification, Enzyme characterization, Cyclodextrin production

## Abstract

Cyclodextrin glucanotransferase (CGTase, EC 2.4.1.9) is an unique enzyme capable of converting starch and related substrates into cyclodextrins (CDs). In this paper, we report an one step gel purification method of CGTase from *Bacillus* sp. and later enzyme characterization. The *Bacillus* sp. strain was isolated from a *Colocacia esculenta* rizospheric soil sample and the CGTase production was carried out in alkaline medium (pH=10). The CGTase purification from the culture supernatant was performed by gel filtration. The enzyme was purified in one step with a recovery of 87.3% activity and 40-fold purification for specific enzymatic activity of 2.24 U/mg. Optimal activity was observed at pH 5.0 in citrate-phosphate buffer, and the enzyme retained almost 100 % of its activity between pH 5.5 and 10 after incubation for 1 h at 4°C. The enzyme exhibited maximum activity at 55°C and showed a T_50%_ of 70°C. The ratio of α:β:γ CD formed by the enzyme was 0.74:1:0.61 for soluble starch and 0.29:1:0.85 for cocoyam starch.

## Background

The alkaliphilic bacilli are the best producers of the enzyme cyclodextrin glucanotransferase (CGTase, EC 2.4.1.19). Cyclodextrin glucanotransferase is a multifunctional enzyme which catalyzes four related reactions: cyclizing, coupling, disproportionation, and hydrolysis. By means of the cyclizing activity, CGTase is an unique enzyme capable of converting starch and related substrates into cyclodextrins (CDs) (Jemli et al. 2007).

Cyclodextrins (CDs) are non-reducing cyclic oligosaccharides with the spatial structure of a doughnut. The interior of CDs is hydrophobic and its external surface is hydrophilic. Due to this feature, CDs are able to form inclusion complexes with either organic or inorganic molecules (Abdel et al. 2011). As a result, CDs can change physical and chemical properties of encapsulated guest compounds. Therefore, CDs are becoming increasingly popular and are extensively used in industries such as pharmaceutical, textile, agriculture, cosmetic, chemical and food, where CDs help to increase the solubility and stability, reduce volatility, and improve the control of the release of drugs, and mask odours and tastes (Atanosova et al. 2008, Hamoudi et al. 2011, Marcon et al. 2009, Sian et al. 2005, Wang et al. 2011).

CGTase is an extracellular, inducible enzyme produced only by microbial cells. The roles of CGTase production in microorganisms are still unclear; however, some researchers believe that starch is converted by CGTase into CDs that cannot be used by other competing organisms. In this way, the CDs can be used as substrate by the CGTase producer.

Most bacterial CGTases mainly produce α-CD, β-CD and γ-CD consisting of six, seven, or eight glucose units, respectively. Thus, CGTase is sometimes classified into three different types (α-, β- and γ-CGTase), depending on the major CD produced. CGTases that synthesize predominantly one type of CD have great commercial importance, because separation of one type of CDs from a mixture of products is time-consuming, costly and tedious (Jemli et al. 2007). The yields and ratios of α-, β- and γ-CD produced from starch differ depending on the origin of the CGTase and the environmental conditions (Schmid 1989). For this reason, purification and characterization of new CGTases attract a great attention in the CDs production field. Alkalophilic bacilli have received the major attention for industrial applications because of their high activity over a wide range of pH and temperatures.

The main objective of this study was to purify CGTase from alkalophilic *Bacillus* species isolated from soil and to characterize some relevant properties of the enzyme.

## Result and Discussion

**Figure 1:**
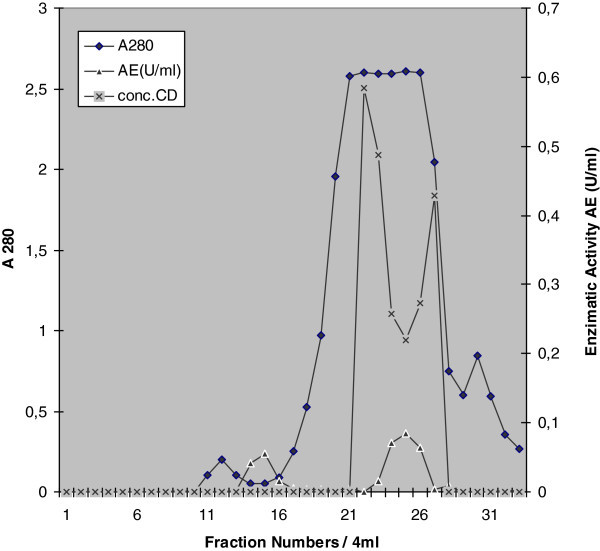
**Elution profile of the CGTase from gel filtration chromatography column using Fractogel EMD BioSEC (S).** Fraction 10–17 was collected and used for subsequent characterization steps.

The CGTase producing strain was identified as Bacillus sp. in previous work Martinez et al. (2012).The CGTase purification from the culture supernatant was performed by gel filtration using Fractogel EMD BioSec(s) as matrix (Figure 1). The active fraction used for the biochemical characterization of the enzyme was located between tube 10 and tube 17, where the enzymatic fraction with the highest purification level was found. In the tube interval from 20 to 30, the concentration in protein (because of the culture medium composition), the cyclodextrin production and the enzymatic activity were maxima, and the enzyme could not be isolated.

The enzyme could be sufficiently purified in one step with a recovery of 87.3% activity and 40-fold purification for specific enzymatic activity of 2.24 U/mg (one unit of enzyme activity refers to the amount of enzyme that catalyzes the production of 1 μmol of β-CD per minute under the reaction condition) (Table 1). A similar result has been reported for *B. agaradhaerens* LS-3C CGTase when sufficiently purified using starch adsorption as the sole purification step with a recovery of 50% activity and 43-fold purification (Martin and Hatti 2002).

**Table 1:** **Summary of CGTase purification results**

Steps	Total volumen (mL)	Protein concentration (mg/mL)	Enzimatic activity (U/mL)	Especific activity. (U/mg)	Purification fold	Yield (%)
Crude enzyme	25	3.1	0.17	0.06	1	100
Concentration	5	15.5	-	-	-	-
Gel filtration (Fractogel)	28	0.06	0.16	2.70	48.2	105.2
Concentration	4.8	0.34	0.77	2.24	40	87.3

**Figure 2:**
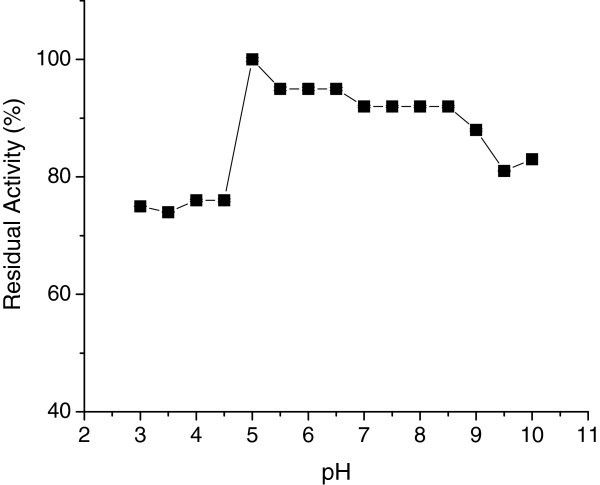
**Optimun pH of CGTase.**

Enzyme activity was measured at 55°C using the standard assay method by varying the pH values from 3.0 to 10.5. The optimum pH of the purified CGTase was 5.0 (Figure 2), which is in agreement with the values reported for purified CGTases from *Bacillu*s sp. 7–12 (Cao et al. 2005) and *B. megaterium* (Pishtiyski et al. 2008). Most CGTase exhibit optimum pH ranging from 5.0 to 8.0 (Sian et al. 2005), although the enzyme from *Brevibacterium* sp. no. 9605 exhibits the highest activity at pH 10.0 (Mori et al. 1994).

**Figure 3:**
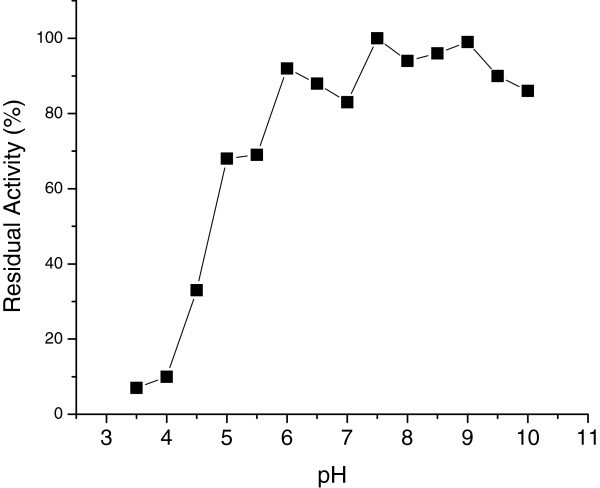
**pH stability of CGTase.**

The enzyme was then incubated for 60 minutes at 4°C under various pH conditions, prior to the determination of residual activity under standard assay conditions. The enzyme retained 85% of its initial activity at pHs between 6 and 10.0. At pH 5.0 the activity retained was 70%; below this pH value a drastic reduction in enzyme activity was observed (Figure 3). When compared to other *Bacillus* CGTases, a similar behavior was observed for *B. agaradhaerens* LS-3C enzyme, which possesses the widest pH span for stability, namely between pH 5.4-11.0 (Martin and Hatti 2002).

**Figure 4:**
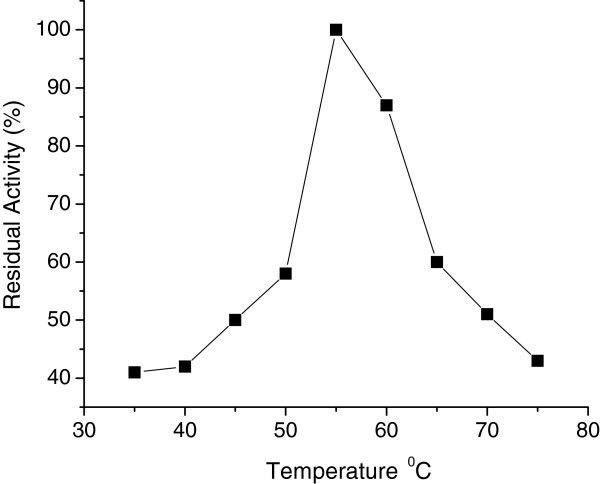
**Optimun temperature of CGTase.**

The activity of the purified CGTase was measured at different temperatures at pH 5.0. As can be seen in Figure 4, the enzyme was optimally active at 55°C when soluble starch was used as substrate. Studies done by other researchers on CGTase from *B. megaterium* (Kitahata et al. 1974), *B. macerans* IFO 3490 (Kaneko et al. 1990), *B. lentus* (Sabioni and Park 1992) and *B. agaradhaerens* (Martin and Hatti 2002) also pointed out 55°C as the optimum temperature. Most of the reported *Bacillus* CGTases shows optimum temperature in the range from 45 to 80°C (Martin and Hatti 2002).

**Figure 5:**
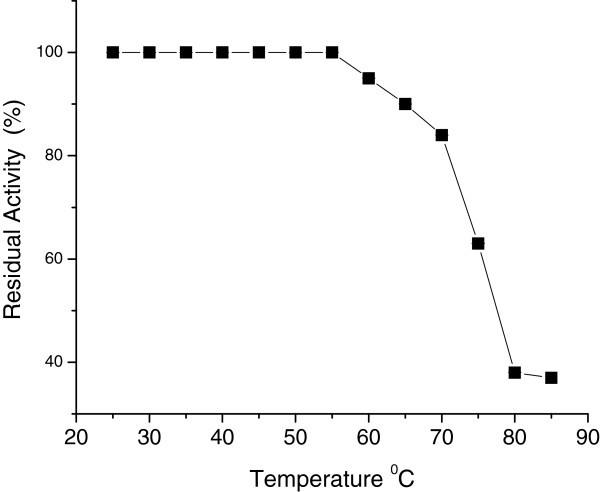
**Thermal stability of CGTase.**

The enzyme was stable up to 55°C at pH 5.0 for 30 min incubation, retaining almost 100% activity. However, it lost 15% of its total activity at around 70°C and had 65% activity at 75°C. The CGTase was completely inactive at 85°C (Figure 5). Thus, the isolated enzyme has higher temperature stability than CGTase from *K. pneumoniae* AS-22 (Gawande and Patkar 2001) and *B. agaradhaerens* (Martin and Hatti 2002), both showing maximum stability at 40°C.

**Figure 6:**
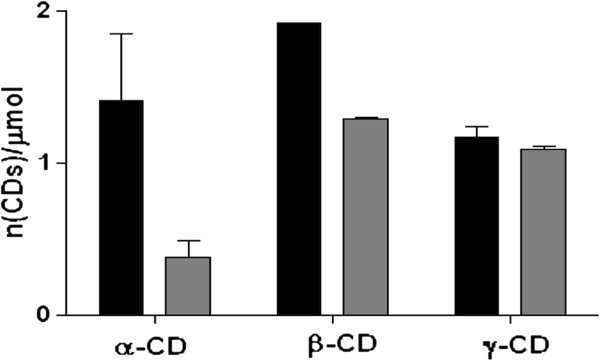
**Production of cyclodextrins on soluble (black) and cocoyam starch (gray).**

The capability of the purified CGTase to produce CDs was investigated using starches from different sources. Soluble starch appeared to be the best substrate for β-CD production, leading to α:β:γ production ratio of 0.74:1:0.61 (Figure 6). Cocoyam starch is a non conventional source to produce CDs; nevertheless, it may have interest as substrate of the isolated enzyme since it caused a marked change in the α:β:γ production ratio, i.e. 0.29:1:0.85, with respect to the soluble starch. Cocoyam starch notably increased the γ-CD production, which has high market price. The α:β:γ proportion obtained for cocoyam starch was similar to that reported for alkalophilic *Bacillus* sp. 7–12 CGTase (α:β:γ = 0.26:1:0.86) with soluble starch after 12 h of reaction at 60°C, pH 8.0 (Cao et al. 2005). These findings confirm that the source of starch is a critical point in the production of CDs, which can be attributed to the differences in the starch granules structure and properties.

## Conclusion

Cyclodextrin glucanotransferase (CGTase) from alkalophilic *Bacillus* has been successfully purified and effectively used for conversion of starch into cyclodextrin under a wide range of pH conditions. The enzyme exhibits a good thermostability and shows optimum temperature at 55°C. Furthermore, it is a good candidate to obtain increased production of γ-CD production when used cocoyam starch as substrate.

## Material and Methods

### Materials

α-, β- and γ-Cyclodextrin were purchased from Pharmacia Biotech. Soluble starch, Fractogel EMD BioSec(s), yeast extract and peptone was purchased from Sigma. Cocoyam starch was of industrial grade. Phenolphthalein was purchased from Merck. All other chemicals were of analytical grade.

### Screening and Isolation of bacteria

Bacteria were isolated from *Colocacia esculenta* rizospheric soil. Samples were suspended in sterile water, serial diluted, and then plated on Horikoshi II agar plate containing (w/v): 1.0 % soluble starch, 0.5 % yeast extract, 0.5 % peptone, 0.1 % KH_2_PO_4_, 0.02 % MgSO_4_.7H_2_O, 0.02 % phenolphthalein and 1.0 % Na_2_CO_3_ (separately autoclaved). Plates were incubated at 37°C for 24 h. Bacterial colonies which produced the largest clear halo zones were selected for further studies (Illias et al. 2002).

### CGTase production and purification

The selected strain was cultivated in flasks containing 200 mL of Horikoshi II broth culture medium and incubated at 37°C during 48 hours at 200 rpm. Cells and insoluble material were removed by centrifugation at 2012 g for 15 min at 4°C, and the cell-free supernatant was used as the source of the enzyme. The crude extract (25 ml) was concentrated to a final volume of 5 ml by rotoevaporation to 30°C. Later, the sample was purified to 4°C in chromatographic column of gel filtration(1.6X 60cm) using Fractogel EMD BioSec(s) as matrix with mobile phase buffer Tris/HCl pH 8.0 20 mM. The column was washed with the same buffer at flow rate 60 mL/h at fractions of 4ml was collected. The protein concentration was estimated using the Bradford method (Bradford 1976).

### Cyclizing activity of CGTase

The cyclizing activity of CGTase was determined applying the phenolphthalein method, measuring the production of β-CD spectrophotometrically at 550 nm, on the basis of its ability to form a colourness inclusion complex with this dye. Phenolphtalein (4 mM) in ethanol was diluted in 125 mM Na_2_CO_3_ pH 11 just before starting the assay. 2% Starch in 0.05 M citrate-phosphate buffer pH 5.0 were used as substrate. One unit of CGTase is defined as the amount of enzyme catalyzing the production of 1 μmol of β-CD per minute under the reaction conditions (Goel and Nene 1995).

### Optimum pH

The effect of pH on CGTase activity was measured in the range from 3.0 to 10.5, at 55°C for 10 minutes using 50mM citrate-phosphate (pH 3.0-7.5), 50mM Tris–HCl (pH 8.0-9.0) and 50mM sodium carbonate (pH 9.0–11) buffers.

### Stability against pH

Enzyme preparations (0.57 U) were incubated at 4°C in 100mM sodium acetate/acetic acid (pH 3.0-5.5), 100mM K_2_HPO_4_/ KH_2_PO_4_ (pH 6.0-7.5), 100mM Tris/HCl (pH 8.0-9.0) and Na_2_CO_3_ /NaHCO_3_ (pH 9.5-11.5) buffers. Aliquots were removed after 60 minutes of incubation, diluted in 0.1 M K_2_HPO_4_/ KH_2_PO_4_ buffer (pH 7.0) and assayed for cyclizing activity of CGTase.

### Optimum temperature

The effect of temperature on CGTase activity was evaluated in the range of 35-75°C in 50mM citrate phosphate (pH 5.0) buffer. After incubation for 10 minutes, the cyclizing activity was measured.

### Thermostability

Enzyme preparations (0.57 U) were incubated at different temperatures between 25°C and 85°C in 50mM sodium citrate phosphate buffer, pH 5.0. Aliquots were removed after 30 minutes incubation, chilled quickly, and assayed for cyclizing activity.

### Determination of CDs concentration

The enzyme (0.27mg/mL) was incubated at 55°C for 60 minutes with 1.5% starch in 50mM citrate phosphate buffer (pH=5.0). The concentration of the various CDs produced by the action of the purified CGTase on soluble and cocoyam starch was colorimetrically determined. The concentration of α-CD was assayed by the decrease in absorbance at 507 nm due to the formation of inclusion complexes with methyl orange (Higuti et al. 2004). The concentration of β-CD was determined according to the method described above (Goel and Nene 1995). The concentration of γ-CD was quantified by measuring the absorbance at 630 nm due to the formation of inclusion complexes with bromocresol green (Kato and Horikoshi 1984).

All experiments were carried out in triplicate under identical conditions.

## Abbreviations

CGTase: Cyclodextrin glucanotransferase
CDs: Cyclodextrins.
